# Investigating the Influence of Impurity Defects on the Adsorption Behavior of Hydrated Sc^3+^ on the Kaolinite (001) Surface Using Density Functional Theory

**DOI:** 10.3390/ma17030610

**Published:** 2024-01-26

**Authors:** Kaiyu Wang, Zilong Zhao, Guoyuan Wu, Dengbang Jiang, Yaozhong Lan

**Affiliations:** 1 School of Materials and Energy, Yunnan University, Kunming 650091, China; 2 Green Preparation Technology of Biobased Materials National & Local Joint Engineering Research Center, Yunnan Minzu University, Kunming 650500, China

**Keywords:** kaolinite, ion-adsorbed rare earths, scandium, impurity ions, DFT

## Abstract

In natural kaolinite lattices, ${Al}^{3+}$ can potentially be substituted by cations such as ${Mg}^{2+}$, ${Ca}^{2+}$, and ${Fe}^{3+}$, thereby influencing its adsorption characteristics towards rare earth elements like ${Sc}^{3+}$. Density functional theory (DFT) has emerged as a crucial tool in the study of adsorption phenomena, particularly for understanding the complex interactions of rare earth elements with clay minerals. This study employed DFT to investigate the impact of these three dopant elements on the adsorption of hydrated ${Sc}^{3+}$ on the kaolinite (001) Al-OH surface. We discerned that the optimal adsorption configuration for hydrated ${Sc}^{3+}$ is ${Sc(H_{2}O)}_{8}^{3+}$, with a preference for adsorption at the deprotonated O_u_ sites. Among the dopants, Mg doping exhibited superior stability with a binding energy of −4.311 eV and the most negative adsorption energy of −1104.16 kJ/mol. Both Mg and Ca doping enhanced the covalency of the Al-O bond, leading to a subtle shift in the overall density of states towards higher energies, thereby augmenting the reactivity of the O atoms. In contrast, Fe doping caused a pronounced shift in the density of states towards lower energies. Compared to the undoped kaolinite, Mg and Ca doping further diminished the adsorption energy of hydrated ${Sc}^{3+}$ and increased its coordination number, while Fe doping elevated the adsorption energy. This study offers profound insights into understanding the role of dopant elements in the adsorption of hydrated ${Sc}^{3+}$ on kaolinite.

## 1. Introduction

Rare earth elements, particularly scandium (Sc), have gained extensive attention from both the scientific and industrial communities due to their unique physical and chemical properties, finding applications in optical, electrical, magnetic, superconducting, catalytic, and other high-tech domains [1,2,3,4]. Ion-adsorption type rare earth ores, serving as the primary source for medium and heavy rare earths, are characterized by the adsorption of rare earth elements predominantly in the form of hydrated or hydroxylated complexes on mineral surfaces [5]. Consequently, conventional ore beneficiation techniques, such as magnetic separation and flotation, are not applicable to these ores. However, effective extraction of rare earths from these ores can be achieved through ion exchange using electrolyte solutions like NH_4_Cl or (NH_4_)_2_SO_4_ [6]. This structure and composition offer novel opportunities for the extraction and utilization of rare earth elements but also present a myriad of challenges. For instance, the ion-exchange method typically necessitates the use of copious chemical reagents, escalating costs and potentially leading to environmental contamination. In-situ leaching is the most prevalent leaching technique, boasting the advantage of eliminating the need for mining and surface disruption, yet it results in ammonia nitrogen pollution [7,8]. Furthermore, issues like the re-adsorption of rare earth elements and suboptimal leaching efficiency persist [9].

**Figure 1:**
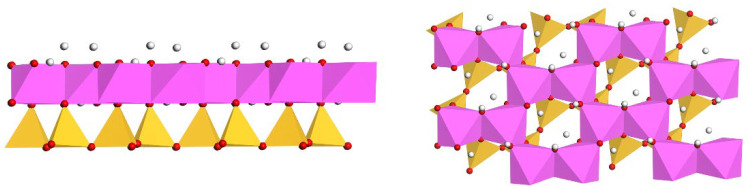
Schematic representation of the kaolinite crystal structure (**Left**: Front view; **Right**: Top view. Purple: Al, Yellow: Si).

Kaolinite is the principal mineral component of ion-adsorption-type rare earth ores [10]. Its structure is composed of alternating silicon-oxygen tetrahedral sheets and aluminum-oxygen octahedral sheets, forming a 1:1 layered silicate structure, as depicted in Figure 1, with these atomic layers stacking along the c-axis [11]. Owing to the interlayer hydrogen bonding in kaolinite, water molecules find it challenging to penetrate between the layers, resulting in kaolinite having a relatively low swelling coefficient. Kaolinite tends to cleave easily along its (001) plane [12]. Due to its exceptional selective adsorption capability, it serves as the primary host for scandium and other rare earth elements in the Earth’s crust. Studies indicate that rare earth elements genuinely adsorb onto kaolinite in the form of easily leachable 8 to 9 coordinated outer-sphere hydrated complexes [13]. Throughout the formation and evolution of the Earth’s crust, natural kaolinite frequently interacts with other minerals and compounds. This interaction leads to the emergence of various defects and impurities within its lattice structure. A common occurrence in this process is the substitution of ${Al}^{3+}$ or ${Si}^{4+}$ positions by other metal cations such as ${Mg}^{2+}$, ${Fe}^{3+}$, or ${Ca}^{2+}$. These substitutions endow the basal surface of kaolinite with permanent negative charge sites and result in notable crystallographic alterations. It’s these alterations that significantly influence the adsorption behavior of kaolinite, especially toward rare earth elements like ${Sc}^{3+}$ [14,15]. The decision to focus our study on the substitution effects of ${Mg}^{2+}$, ${Fe}^{3+}$, and ${Ca}^{2+}$ for ${Al}^{3+}$ is driven by their geochemical relevance and prevalence in natural kaolinite formations. By investigating these specific elemental substitutions, we aim to gain a more accurate understanding of the adsorptive interactions in natural scenarios, offering crucial insights into the complex mechanisms governing the adsorption of rare earth elements on kaolinite surfaces.

While numerous experiments have been conducted to elucidate the adsorption characteristics of rare earth elements on kaolinite surfaces, the microscopic mechanisms remain somewhat nebulous. Most extant research is predicated on simplified models, potentially overlooking the intricacies inherent under genuine conditions, especially the ramifications of impurity defects like ${Mg}^{2+}$ within kaolinite. Against this backdrop, employing first principles to study the adsorption of rare earth elements on kaolinite surfaces becomes paramount. Through such theoretical computational methods, we can delve into the adsorption mechanisms of rare earth elements on kaolinite surfaces, furnishing theoretical underpinning for devising novel rare earth extraction techniques, thereby enhancing leaching efficiency and mitigating environmental contamination. Presently, density functional theory (DFT) has been extensively adopted in studies concerning the adsorption of rare earths and other substances on clay minerals. For instance, Qiu et al. [16] employed density functional theory (DFT) simulations to explore the adsorption behavior and bonding mechanisms of yttrium complexes on kaolinite (001) surfaces. They discovered that the yttrium complex ${{Y(OH)}_{2}(H_{2}O)}_{7}^{+}$, with its lowest binding energy in an aqueous system, preferred adsorption on the Al-OH surface through coordination bonds. This study highlighted the significant influence of steric hindrance on adsorption preferences, providing critical insights into the adsorption mechanisms of rare earth elements on clay minerals. Santana et al.’s [17] study focuses on the adsorption of 4-nitrophenol onto kaolinite using DFT with van der Waals functionals. They found that 4-nitrophenol adsorbs effectively through hydrogen bonds, highlighting kaolinite’s potential as a low-cost adsorbent for contaminants. El Hassani et al. [18] investigated the adsorption of Rhodamine B dye onto kaolinite and hydroxyapatite in aqueous solutions, combining molecular dynamics simulation, DFT calculations, and experimental study. They optimized adsorption conditions using the Box-Behnken design and found high dye removal efficiencies with kaolinite and hydroxyapatite. Kremleva et al. [19] employed DFT to investigate the adsorption of uranyl ions on kaolinite basal planes, revealing that adsorption complexes formed with deprotonated OH groups on the Al(o) surface exhibited superior adsorption energies. Kasprzhitskii et al.’s [20] DFT study focuses on aliphatic amino acids’ adsorption mechanisms on kaolinite surfaces, emphasizing the key role of amino acids’ carboxyl groups in hydrogen bonding interactions. Lainé et al. [21] explored the adsorption of phenol, toluene, ${CO}_{2}$, and water on kaolinite using density functional theory and ab initio molecular dynamics. They found that dispersion interactions significantly influence the adsorption on kaolinite’s siloxane surface. The study revealed that phenol and toluene strongly adsorb on the aluminol surface of kaolinite due to hydrogen bonding and π-interactions, suggesting kaolinite’s potential to remove these compounds from the air, particularly phenol. This research enhances the understanding of molecular interactions in adsorption processes on clay minerals. García et al. [22] used molecular dynamics simulations to study phosphate ion adsorption on kaolinite surfaces in low-salt solutions. They examined adsorption on kaolinite’s basal and edge surfaces, finding that phosphates preferentially form large clusters for adsorption, especially on hydrophilic surfaces. Salt presence enhances phosphate adsorption through cationic bridges.

Historical research predominantly focused on the adsorption of rare earth cations on ideal clay mineral surfaces under vacuum conditions [23,24]. However, kaolinite in real-world scenarios is often influenced by a plethora of impurities, which can significantly modulate its adsorption performance towards rare earth ions. Notably, scandium (Sc), owing to its unique properties and scarcity in high-tech applications, has emerged as a focal point in rare earth element research. In contrast to the majority of previous studies, our research specifically investigates the substitution effects of ${Mg}^{2+}$, ${Ca}^{2+}$, and ${Fe}^{3+}$ ions for ${Al}^{3+}$ in the kaolinite structure, focusing on the resulting changes in adsorption behavior towards ${Sc}^{3+}$. Furthermore, we also consider the hydrated configurations of ${Sc}^{3+}$, which adds another layer of complexity and realism to our study. This aspect has not been thoroughly explored in the existing literature, making our study a significant contribution to the field. It not only enhances our understanding of impurity impacts in realistic environmental conditions but also bridges theoretical insights with practical applications, specifically in refining strategies for the extraction and recovery of rare earth elements. Utilizing density functional theory, our study aims to guide experimental research, fostering advancements in the field of adsorption technology.

## 2. Theoretical Methods and Models

### 2.1. Calculation Methods and Parameters

All computational endeavors were executed within the framework of density functional theory (DFT) utilizing the CASTEP 19.1 software package [25,26,27,28]. The generalized gradient approximation (GGA) was employed, incorporating the Perdew-Burke-Ernzerhof (PBE) function [29]. To aptly simulate van der Waals interactions inherent in real systems, the Grimme correction was integrated [30]. A 2 × 2 × 1 supercell was adopted for the kaolinite surface structure, with cell parameters derived from our prior optimized work [31]: a = 5.184 Å, b = 8.983 Å, c = 7.347 Å, α = 91.48°, β = 105.01°, and γ = 89.91°, exhibiting a discrepancy of less than 2% compared to experimental values [32]. To ensure computational stability and precision, convergence tests were meticulously conducted for pivotal parameters such as the cutoff energy and k-point grid. The cutoff energy was designated at 480 eV. During bulk phase optimization, a (3 × 2 × 2) Monkhorst-Pack k-point grid was employed, while a (3 × 2 × 1) k-point grid was harnessed for surface and adsorption system optimization [33]. The energy convergence criterion for the self-consistent field (SCF) was set at 2 × 10^−6^ eV/atom. Geometric optimization convergence criteria were delineated as maximum atomic force 0.03 eV/Å, maximum stress 0.05 GPa, and maximum atomic displacement 0.001 Å. The Broyden–Fletcher–Goldfarb–Shanno (BFGS) algorithm was employed for geometric optimization [34]. Ultrasoft pseudopotentials were utilized [35], with valence electron configurations specified as H 1s^1^, O 2s^2^2p^4^, Al 3s^2^3p^1^, Si 3s^2^3p^2^, Fe 3d^6^4s^2^, Mg 3s^2^, Ca 4s^2^, and Sc 3d^1^4s^2^. For systems doped with Fe, spin polarization was activated, and the DFT+U method was implemented, with the Hubbard U value designated at 4 eV [14,36]. Through this array of optimized computational parameters and methodologies, we were not only able to accurately simulate the adsorption behavior of hydrated ${Sc}^{3+}$ on kaolinite surfaces but also gain profound insights into how impurities and defects modulate this process.

### 2.2. Model Construction

In this study, we extended our prior investigations on the kaolinite (001) surface, which exposes the Al-OH layer. This surface model encompasses six atomic layers, delineated as “H-O-Al-O-Si-O” [18]. To simulate authentic adsorptive conditions, atomic constituents within the basal “O-Si-O” tier were anchored, allowing solely the superior “H-O-Al” atomic strata to undergo relaxation. To obviate potential deleterious interactions stemming from periodic boundary stipulations, a vacuum layer of 15 Å was instituted atop the surface.

**Figure 2:**
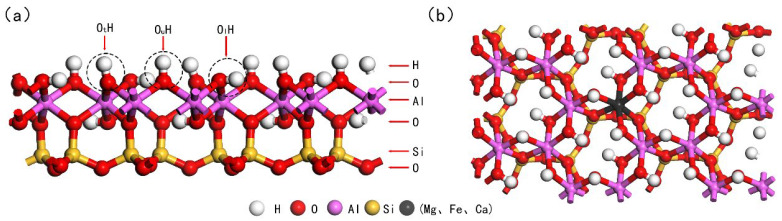
Optimized structure of the kaolinite (001) surface ((**a**): Front view after optimization; (**b**): Top view post-doping).

In our study, we focused on the effects of doping elements Mg, Ca, and Fe, which substitute the central Al atom in the Al-OH surface of kaolinite. We observed that when the environmental pH value exceeds 5.5 [19], surpassing the point of zero charge (PZC) of kaolinite in water, the kaolinite surface undergoes deprotonation, acquiring a negative charge. This enhances its adsorptive capacity for rare earth ions. Upon optimization, the kaolinite (001) Al-OH surface exhibited three distinct hydroxyl groups: tilted hydroxyl groups (O_t_H), lying hydroxyl groups (O_l_H), and upright hydroxyl groups (O_u_H) [37]. This proffers an array of prospective adsorptive loci for ensuing adsorptive explorations, as illustrated in Figure 2. Figure 2a showcases the front view of the optimized kaolinite (001) surface, while Figure 2b presents the top view of the doped kaolinite (001) surface. To gauge the stability of the doped surfaces, we computed the binding energies ($E_{b}$) for various doped systems. The binding energy is defined as [22]:

(1) $$E_{b}=\frac{\left(E_{s-D}-\sum_{i}N_{i}E_{i}\right)}{\sum_{i}N_{i}}$$

In the aforementioned equation, $E_{s-D}$ represents the total energy of the kaolinite (001) surface with impurity defects. The symbol $N_{i}$ denotes the number of Al, Si, O, H, and doped atoms in the kaolinite surface structure, while $E_{i}$ corresponds to the energy of these individual atoms. It’s salient to highlight that within the 2 × 2 × 1 supercell, a total of 136 atoms are encompassed. The binding energy, $E_{b}$ emerges as a fundamental metric for gauging the stability of the doped surface. Explicitly, a more negative $E_{b}$ value implies the augmented stability of the doped surface. This proffers an efficacious quantitative methodology to discern the ramifications of diverse impurity elements on the stability of the kaolinite surface.

Based on prior research [31], the optimal adsorption configuration for hydrated ${Sc}^{3+}$ is ${Sc(H_{2}O)}_{8}^{3+}$, with a binding energy of −2629.44 kJ/mol. To further investigate its adsorption characteristics on the kaolinite (001) Al-OH surface, we initially removed one coordinating water molecule. Subsequently, it was positioned near the central Al atom of the kaolinite (001) Al-OH surface, adjacent to three deprotonated oxygen atoms (corresponding to the lying oxygen O_l_, tilted oxygen O_t_, and upright oxygen O_u_, respectively) as the initial adsorption configuration. The adsorption energy ($E_{ads}$) was employed to assess the stability of hydrated ${Sc}^{3+}$ adsorbed on the kaolinite (001) Al-OH surface, and its calculation formula is:

(2) $$E_{ads}=E_{Sc/c}-E_{Sc}-E_{s}$$

In this equation, $E_{Sc/s}$ represents the total energy of the adsorption complex formed between the hydrated ${Sc}^{3+}$ and the kaolinite (001) surface. $E_{s}$ is the total energy of the kaolinite (001) surface prior to the adsorption of hydrated ${Sc}^{3+}$, and $E_{Sc}$ denotes the total energy of the hydrated ${Sc}^{3+}$. The magnitude of the adsorption energy reflects the stability of the species adsorbed on the kaolinite (001) surface and indicates the preferred adsorption site of the hydrated ${Sc}^{3+}$. Lower adsorption energy typically signifies that the system has reached a more energetically favorable state upon the formation of the adsorption complex, thereby enhancing the stability of the adsorbed species.

After the optimization of the kaolinite (001) Al-OH surface, to ensure that our models were not trapped in local minima and truly represented the minimal energy state for each system, we employed a strategy of constructing multiple initial configurations. For each model, five different initial structures were created and evaluated. The structure with the lowest energy among these was then selected for further computational analysis. This method allowed us to compare and choose the most stable configuration, providing a more reliable assessment of the adsorption properties.

## 3. Results and Discussion

### 3.1. Structure and Properties of the Adsorption Model ${Sc(H_{2}O)}_{8}^{3+}$

**Figure 3:**
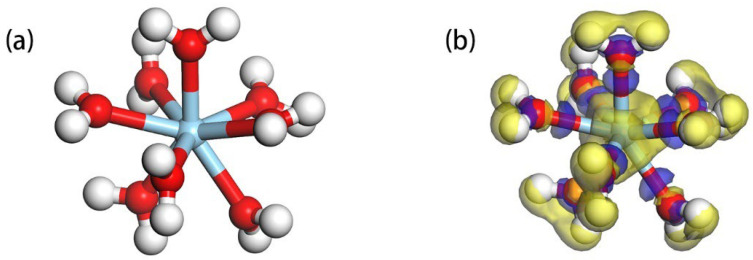
(**a**) Equilibrium geometric configuration of ${Sc(H_{2}O)}_{8}^{3+}$; (**b**) Charge density difference of ${Sc(H_{2}O)}_{8}^{3+}$ relative to ${Sc}^{3+}$ (The isosurface value is 0.05, The blue sphere represent Sc).

Considering the crucial role of hydration in the adsorption process, we have chosen ${Sc(H_{2}O)}_{8}^{3+}$ as our adsorbate model. This selection aids in simulating the hydrated state in real environments more accurately, thereby offering a more genuine depiction of the adsorption and reaction mechanisms. Figure 3 presents the (a) equilibrium geometric configuration and (b) differential charge density plot of ${Sc(H_{2}O)}_{8}^{3+}.$ The latter is employed to display variations in electronic density within the structure, assisting in understanding electron migration and the formation of chemical bonds. As shown in (a), the eight water molecules are evenly distributed around the Sc ion, establishing a highly symmetrical hydration structure that contributes to the overall stability. In (b), the yellow and blue regions respectively denote areas of electron loss and gain. Evidently, H and Sc are situated in electron-depleted regions, bearing positive charges, while O atoms, due to their high electronegativity, are located in electron-rich areas, carrying negative charges. Table 1 meticulously enumerates the Mulliken atomic charge population of O and Sc in ${Sc(H_{2}O)}_{8}^{3+}$ and the bond lengths of Sc-O and their charge population. Table 1 indicates that the charge on O transitions from −2 to a range of −0.94 to −0.98, while the charge on Sc shifts from 3 to 2.04. The bond lengths of Sc-O span from 2.181 Å to 2.357 Å, with bond population values ranging between 0.13 and 0.14. This suggests a pronounced electron transfer between Sc and the surrounding O, with the Sc-O bond exhibiting significant ionic character, corroborating the observations in Figure 3.

### 3.2. Properties of Ideal and Doped Kaolinite (001) Al-OH Surfaces

Before proceeding with the calculation of binding energies, comprehensive structural optimizations were performed for kaolinite (001) surfaces doped with Mg, Ca, and Fe. These optimizations utilized the same parameters as those applied to the pristine kaolinite surface. Subsequently, the binding energies for these three distinct doping systems were determined, with the detailed outcomes delineated in Table 2. All three dopings (Mg, Ca, Fe) yielded negative $E_{b}$ values, indicating that doping enhances the stability of the kaolinite surface. Among them, the Mg-doped kaolinite surface exhibits the highest stability with an $E_{b}$ value of −4.311 eV/atom, followed by Ca doping (−4.286 eV/atom), and lastly, Fe doping (−4.179 eV/atom). This result is consistent with the fact that natural kaolinite contains a higher content of Mg [38].

**Figure 4:**
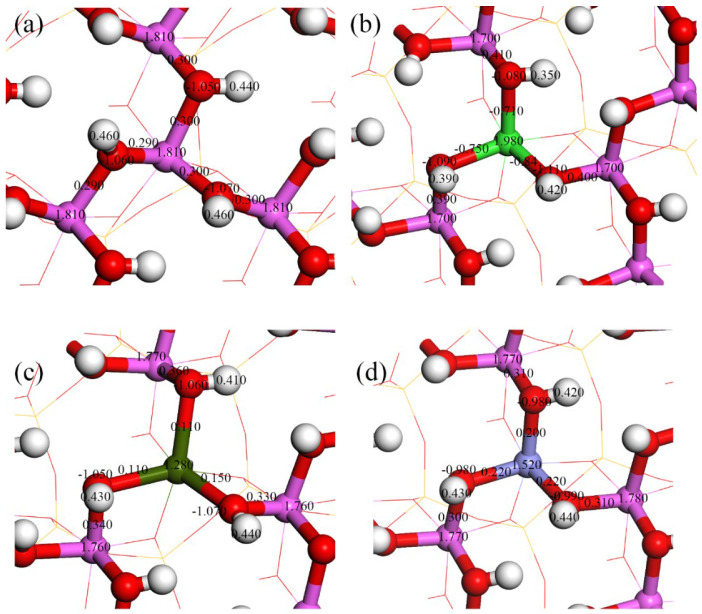
Mulliken atomic and bond populations of the ideal and doped kaolinite (001) Al-OH surfaces ((**a**) ideal kaolinite surface; (**b**) Mg-doped kaolinite surface; (**c**) Ca-doped kaolinite surface; (**d**) Fe-doped kaolinite surface).

As a common clay mineral, kaolinite’s surface properties are crucial in adsorption behaviors. Different doping elements significantly affect the surface’s atomic charges and chemical bonds [39,40]. Through the Mulliken population analysis, we analyzed in detail the effects of Mg, Ca, and Fe doping on surface properties, as shown in Figure 4. Without doping (Figure 4a), the charges on Al and oxygen O on the kaolinite surface are approximately 1.81 and −1.05, respectively, and the population value of the Al-O bond is about 0.30. On the Mg-doped kaolinite surface (Figure 4b), the Mg atom has a charge of 1.98, and the charge on the O atom changes to about −1.10. The Mulliken population values of the Mg-O bond range from −0.84 to −0.71, and such negative values typically imply antibonding properties. However, in this case, this negative value complements the influence of Mg on the charges of surrounding Al and H atoms, leading to an overall increase in surface stability. Specifically, the inclusion of Mg reduces the charge on the adjacent Al atom from 1.77 to 1.70 and increases the population value of the Al-O bond from 0.30 to about 0.40, enhancing the covalency of the Al-O bond. In the case of Ca doping (Figure 4c), the charge on Ca is 1.28, with the Ca-O bond population value dropping to 0.11. This might be due to the lower electronegativity of Ca, which has a greater difference from the electronegativity of O. Furthermore, the atomic populations of O and H remain almost unchanged, indicating that the inclusion of Ca has little effect on the charge distribution of these atoms. The population values of the adjacent three Al atoms decrease from around 1.81 to 1.76, and the population values of the associated Al-O bonds slightly increase from 0.30 to 0.33, 0.34, and 0.36. This suggests that the inclusion of Ca slightly enhances the covalency of the Al-O bond, but this change is much less pronounced than in the case of Mg doping. Figure 4d shows the case of iron doping, where the charge on iron is 1.52, with the iron-oxygen bond population values at 0.20, 0.22, and 0.22. Iron doping has a certain influence on the charge distribution of surrounding atoms; the charge on the O atom changes from −1.05 to −0.98, and the charge on the H atom also decreases from 0.46 to 0.43. The charge on the adjacent Al atom decreases from 1.81 to 1.77, but the population value of the Al-O bond remains almost unchanged, still around 0.30. Overall, the influence of iron doping on the kaolinite surface is relatively minor.

**Figure 5:**
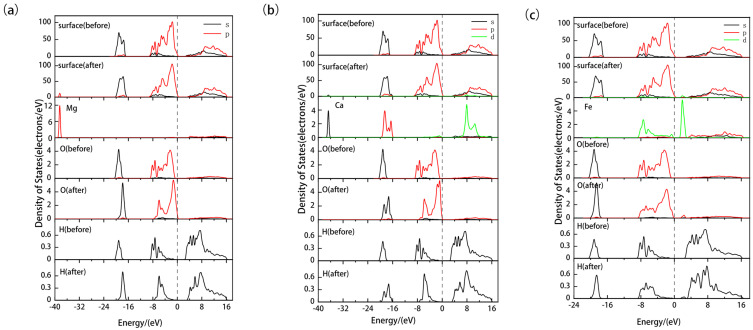
Partial density of states of doped kaolinite (001) Al-OH surfaces ((**a**) Mg-doped kaolinite surface; (**b**) Ca-doped kaolinite surface; (**c**) Fe-doped kaolinite surface).

Analysis of the density of states is crucial for understanding the electronic structural properties of materials. We paid particular attention to the influence of doping on the electronic properties of the kaolinite surface. As shown in Figure 5a–c, we present the partial density of states of the kaolinite surface before and after Mg, Ca, and Fe doping. For Mg doping, Figure 5a shows a slight shift of the overall density of states towards the higher energy direction, which might be related to the increase in surface activity. The peak of Mg 2p orbitals appears around −38 eV, suggesting that Mg doping has a minor influence on the band structure of kaolinite. At the same time, the peaks in the density of states of O and H atoms adjacent to Mg shift overall towards the higher energy direction, and the peak of the 2p orbital of the O atom shifts towards the Fermi level. Their respective density of states peaks also show a significant increase in localization, implying that the electron density is more concentrated on these atomic orbitals. For Ca doping, Figure 5b displays its influence on the density of states of the kaolinite surface, similar to Mg, causing the overall density of states to shift slightly towards the higher energy direction. However, the energy levels of Ca 4s and 3d orbitals are close, introducing a new 3d orbital peak above the Fermi energy level, altering the original band structure, and providing a new electron transport path. The non-locality of the valence band O 2s, 2p orbitals, and H 1s orbitals is enhanced after doping. For Fe doping, Figure 5c shows that it causes a significant shift of the overall density of states towards the lower energy direction, suggesting enhanced stability of the surface. Additionally, the main contribution at the Fermi level transitions from O 2p orbitals to Fe 3d orbitals, indicating a change in the nature of surface interactions due to altered electronic structure.

The investigation of Mg, Ca, and Fe doping on the kaolinite (001) Al-OH surface elucidated distinct impacts of each element on the surface properties. Binding energy analysis shows that Mg doping provides the best stability, followed by Ca, and Fe has the lowest stability. Mg doping enhances the activity and stability of the surface, especially strengthening the covalency of the Al-O bond, and slightly shifting the density of states towards the higher energy direction. The contribution of the 2p orbitals at the Fermi level also increases. The influence of Ca doping on surface properties is similar to that of Mg, but the effect is relatively weaker. Fe doping has little influence on the surrounding Al-O bonds, mainly causing a significant shift of the density of states towards the lower energy direction and shifting the main contribution at the Fermi level from O 2p orbitals to Fe 3d orbitals.

### 3.3. Adsorption of Hydrated ${Sc}^{3+}$ on the Ideal Kaolinite (001) Al-OH Surface

**Figure 6:**
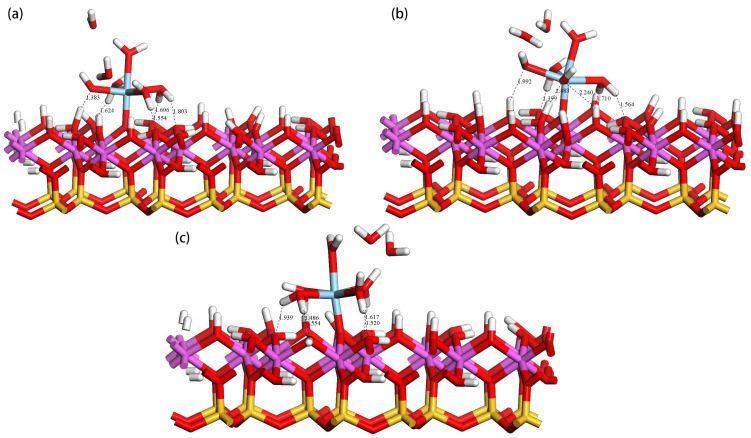
Adsorption configurations of hydrated ${Sc}^{3+}$ at the O_u_ (**a**), O_t_ (**b**), and O_l_ (**c**) sites.

On the deprotonated (001) Al-OH surface of kaolinite, the hydrated ${Sc}^{3+}$ primarily occupies three adsorption sites: tilted hydroxyl groups (O_t_H), lying hydroxyl groups (O_l_H), and upright hydroxyl groups (O_u_H). Through DFT calculations, we found that the hydrated ${Sc}^{3+}$ forms a monodentate adsorption structure with the deprotonated oxygen atoms on the surface, as shown in Figure 6. Panels (a), (b), and (c) represent the adsorption configurations at the O_u_H, O_t_H, and O_l_H sites, respectively. In these three configurations, two water molecules dissociate from the hydration shell of ${Sc}^{3+}$ and become free water molecules. This occurs because, as the hydrated ${Sc}^{3+}$ approaches the kaolinite surface, water molecules weakly bound to the ${Sc}^{3+}$ ion are expelled due to steric hindrance. The stability of these adsorption configurations is primarily determined by the monodentate coordination bond and multiple hydrogen bonds. ${Sc}^{3+}$ forms a coordination bond with the deprotonated oxygen atom (O_s_) on the surface, which is the main contributor to the adsorption stability. Simultaneously, the water molecules in the hydrated ${Sc}^{3+}$ form hydrogen bonds with the hydroxyl groups on the surface. These hydrogen bonds are mainly of two types: one formed between the oxygen atom (O_s_) on the kaolinite surface and the hydrogen atom (H_w_) in the hydrate (O_s_-H_w_), and another formed between the oxygen atom (O_w_) in the hydrate and the hydrogen atom (H_s_) on the kaolinite surface (O_w_-H_s_), further enhancing the stability of the adsorption complex. Table 3 lists the adsorption energies and structural parameters in detail. According to the data, the O_u_H site has the most negative adsorption energy of −986.56 kJ/mol, making its adsorption structure the most stable. This conclusion is supported by bond length data, where the average bond length of the Sc-O_w_ bond at the O_u_H site is the shortest, and the bond length of the bond formed between the ${Sc}^{3+}$ ion and the deprotonated oxygen atom on the surface (Sc-O_s_) is also the shortest, at 1.965 Å. The hydrated ${Sc}^{3+}$ forms 5 hydrogen bonds with the surface, with shorter bond lengths, indicating stronger interactions between them.

**Figure 7:**
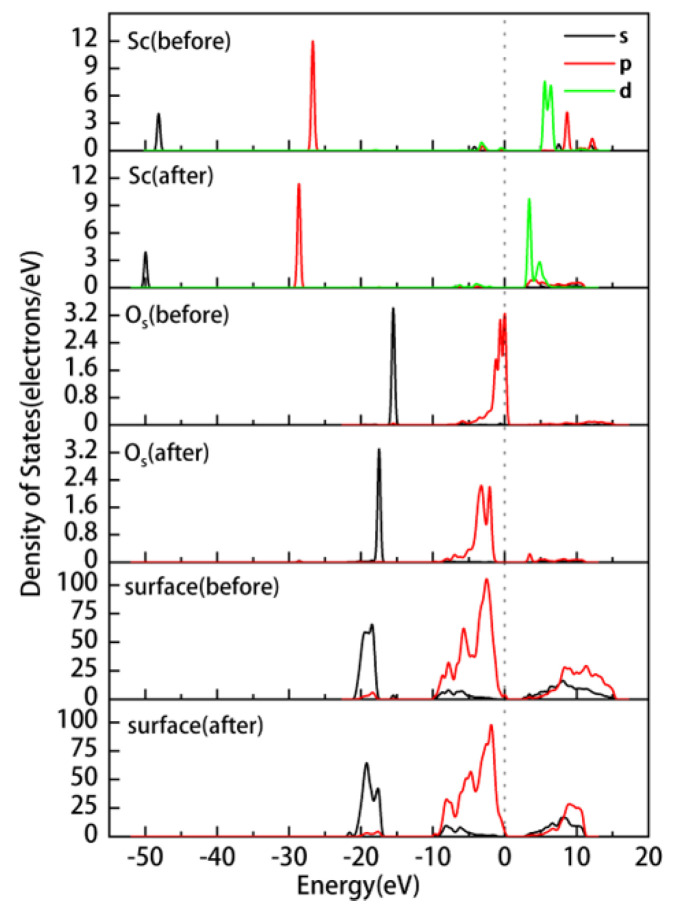
Partial density of states of hydrated ${Sc}^{3+}$ on the kaolinite (001) surface at the O_u_H site before and after adsorption.

We conducted an in-depth analysis of the preferred adsorption site, O_u_H, for hydrated ${Sc}^{3+}$ on the kaolinite surface. Figure 7 displays the partial density of states (PDOS) of ${Sc}^{3+}$, O_s_, and the kaolinite (001) surface before and after adsorption. It is evident from the figure that after deprotonation, the electronic states of the 2p orbitals of O_s_ become more active near the Fermi level. After adsorption, the electronic states near the Fermi level are mainly contributed by the 3d orbitals of Sc and the 2s and 2p orbitals of O_s_, with a noticeable orbital overlap between them. This overlap results in electron transfer from O_s_ to Sc, forming a strong covalent bonding interaction. Additionally, the electronic states of Sc and O_s_ shift significantly towards the lower energy region, reflecting the enhanced stability of the system after adsorption. The overall PDOS of the surface shows increased localization above the Fermi level, indicating a more concentrated energy distribution of these states. Table 4 lists the Mulliken atomic and bond populations of Sc and O_s_ before and after adsorption. The 3p orbitals of Sc gain 0.02e, the 3d orbitals gain 0.12e, and the overall gain is 0.14e, with the charge changing from 2.04 to 1.90. The 2s orbitals of O_s_ lose 0.01e, the 2p orbitals lose 0.20e, and the overall loss is 0.21e, with the charge changing from −1.21 to −1.00. In conjunction with the DOS plot, it can be inferred that Sc gains electrons from O_s_, leading to an increase in its electronic state density, while the electronic state density of O_s_ decreases. This charge redistribution is primarily due to the hybridization between the 3d orbitals of Sc and the 2s and 2p orbitals of O_s_, resulting in a strong covalent interaction, with a Mulliken population value of 0.45 for the Sc-O_s_ bond. The formation of this covalent bond further strengthens the bonding between atoms, making the system more stable.

### 3.4. Adsorption of Hydrated ${Sc}^{3+}$ on Doped Kaolinite (001) Al-OH Surface

**Figure 8:**
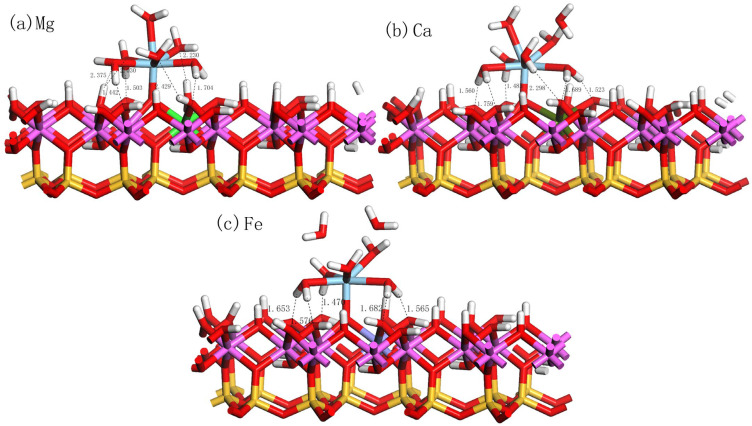
Equilibrium adsorption configurations of hydrated ${Sc}^{3+}$ on the doped kaolinite (001) Al-OH surface at the optimal adsorption site.

Doping is considered a crucial process in kaolinite research, which can significantly influence the physical and chemical properties of kaolinite. Specifically, through doping, the electronic properties of the kaolinite surface can be adjusted, thereby affecting its adsorption performance for various substances, such as metal ions and organic molecules. We found that the adsorption of hydrated ${Sc}^{3+}$ on the doped kaolinite (001) Al-OH surface differs from its adsorption on the undoped kaolinite surface. Figure 8a–c display the optimal adsorption equilibrium configurations of hydrated ${Sc}^{3+}$ on the Mg-, Ca-, and Fe-doped kaolinite (001) Al-OH surface at the O_u_H site. The adsorption energies and structural parameters are shown in Table 5. By comparing the data of different doping elements, we found that Mg and Ca, as divalent cations, introduce negative charges on the kaolinite (001) surface when replacing ${Al}^{3+}$, thereby enhancing the adsorption interaction with positively charged species. This also explains why Mg and Ca doping can enhance the adsorption of hydrated ${Sc}^{3+}$. On the other hand, Fe, as a trivalent cation, has a smaller impact on the adsorption performance of kaolinite.

On the Mg-doped kaolinite surface, we observed that the adsorption behavior of hydrated ${Sc}^{3+}$ is particularly prominent. As seen in Table 5, its water coordination number increases from 5 on the ideal kaolinite surface to 6, and the Sc-O_s_ bond length becomes shorter, implying a more intimate interaction of hydrated ${Sc}^{3+}$ with the Mg-doped surface. Its adsorption energy reaches −1104.16 kJ/mol, the lowest value among the four cases. In contrast, although the Ca-doped kaolinite surface also exhibits good adsorption properties, its adsorption energy is −1088.16 kJ/mol, still higher than the Mg-doped case. The adsorption energy on the Fe-doped kaolinite surface further increases to −853.92 kJ/mol, indicating that Fe doping is not favorable for the adsorption of hydrated ${Sc}^{3+}$.

From the structural parameters, on the Mg-doped kaolinite surface, the average Sc-O_w_ bond length is 2.319 Å, while the Sc-O_s_ bond length is 1.873 Å. This is consistent with its high adsorption stability, indicating a more intimate interaction between Sc and the surface oxygen atoms. Furthermore, the number of hydrogen bonds formed on the Mg-doped kaolinite surface is the highest, with H_s_-O_w_ bond lengths ranging from 2.030 Å to 2.429 Å, and H_w_-O_s_ bond lengths ranging from 1.442 Å to 1.704 Å. On the Ca-doped kaolinite surface, the average Sc-O_w_ bond length is 2.310 Å, and the Sc-O_s_ bond length is 1.869 Å. The number of hydrogen bonds formed also increases, but all hydrogen bonds are of the H_w_-O_s_ type, with bond lengths ranging from 1.485 Å to 2.298 Å, indicating weaker hydrogen bonds compared to the Mg-doped kaolinite surface. On the Fe-doped kaolinite surface, the average Sc-O_w_ bond length is 2.262 Å, and the Sc-O_s_ bond length is 1.971 Å. At the same time, the number of hydrogen bonds formed is the same as that on the pure kaolinite surface, all being of the H_w_-O_s_ type, with bond lengths ranging from 1.470 Å to 1.682 Å, indicating weaker hydrogen bonds compared to the Mg and Ca doped kaolinite surfaces.

**Figure 9:**
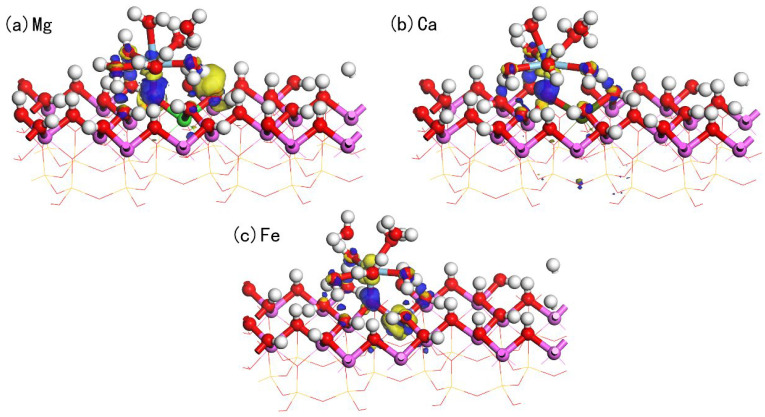
The charge density difference of hydrated ${Sc}^{3+}$ adsorption on the doped kaolinite surface (The isosurface value is 0.05).

To gain a deeper understanding of the interactions between the adsorbate and the adsorbent, we studied the charge density difference and Mulliken charge population of adsorption on the doped kaolinite surface. Figure 9 displays the charge density difference for hydrated ${Sc}^{3+}$ adsorption on three different doped (Mg, Ca, Fe) surfaces, where the yellow regions indicate electron accumulation and the blue regions indicate electron loss. It is evident from the figure that the charge transfer on the Mg-doped surface is the most pronounced, with the largest isosurface. This implies that the charge transfer between the Mg-doped surface and the hydrated ${Sc}^{3+}$ is the most significant, indicating that Mg doping can enhance the interaction between Sc and the surface O_s_ atoms. The prominent yellow region around Sc indicates that Sc loses a specific number of electrons during the adsorption process. Meanwhile, the distinct blue region around O_s_ indicates electron accumulation at these positions, further confirming the charge transfer from Sc to the surface. Additionally, significant charge transfer is observed at the positions where hydrogen bonds are formed.

Table 6 describes the charge distribution of Sc and O_s_ on the ideal and three different doped (Mg, Ca, Fe) surfaces. On the Mg-doped surface, compared to the pure surface, the s-orbital charge of Sc slightly increases from 2.18 to 2.20; the p-orbital charge decreases from 5.90 to 5.88; while the d-orbital charge increases from 1.02 to 1.22. For the O_s_ atom, the s-orbital charge increases from 1.86 to 1.87, while the p-orbital charge decreases from 5.14 to 5.09; the bond population of Sc-O_s_ increases from 0.45 to 0.60. This suggests that Mg doping significantly enhances the interaction between Sc and O_s_. Ca doping has a similar effect on charge population as Mg doping, but its impact is slightly less than that of Mg doping. In contrast, the influence of Fe doping on the charge distribution of Sc and O_s_ is minimal, almost similar to charge distribution when adsorbed on the ideal kaolinite surface, implying that Fe doping does not significantly alter the interaction between Sc and O_s_.

**Figure 10:**
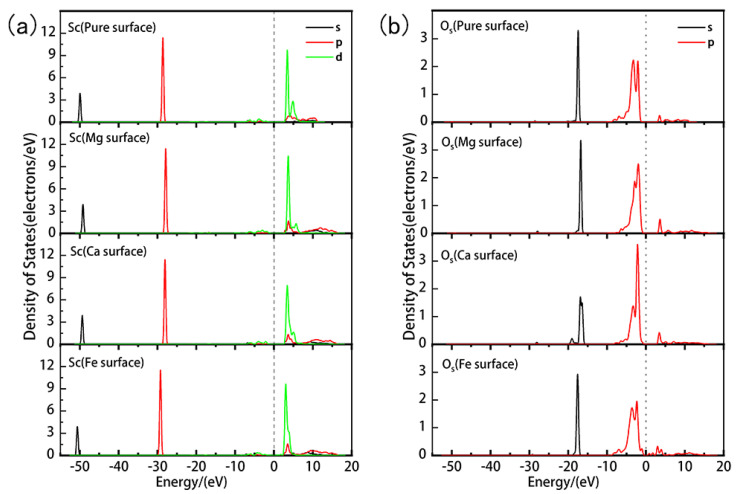
Partial density of states of Sc and O_s_ after adsorption of hydrated ${Sc}^{3+}$ on the ideal and doped kaolinite surfaces. ((**a**) represents the DOS of Sc, (**b**) represents the DOS of O_s_).

In Figure 10, we conducted a detailed analysis of the partial density of states (PDOS) for Sc and O_s_ in different adsorption systems. The results show that Mg and Ca doping lead to a shift of the overall density of states peaks for Sc and O_s_ towards the higher energy direction by approximately 0.7 eV and 0.5 eV, respectively. Such a shift towards the higher energy direction is typically associated with enhanced reactivity, which might explain why the adsorption energies are lower (indicating more stable adsorption) in these two doped systems. Especially in the Mg-doped system, the shift in the density of the state peak is more pronounced, further confirming the enhanced adsorption effect due to Mg doping. While Ca doping also shows an enhanced adsorption effect, its impact is slightly less than that of Mg doping. In contrast, Fe doping results in a shift of the density of states peak towards the lower energy direction by about 0.6 eV and causes the merging of the two 3d peaks in Sc. This shift towards the lower energy direction is typically associated with reduced reactivity, which might be the reason for the weakened adsorption interaction between Sc and O_s_ due to Fe doping.

Based on the above analysis, we find that doping is a crucial factor that can significantly adjust the electronic properties of the kaolinite surface, thereby affecting its adsorption performance for hydrated ${Sc}^{3+}$. Especially for Mg and Ca doping, they enhance the adsorption interaction with positively charged species, with Mg doping performing particularly well, making the adsorption more stable and tighter. In contrast, Fe doping has an adverse effect on the adsorption interaction.

## 4. Conclusions

Through first-principles calculations, this study delves into the influence of three doping elements, Mg, Ca, and Fe, on the adsorption behavior of hydrated ${Sc}^{3+}$ on the kaolinite (001) Al-OH surface. We discovered the optimal adsorption configuration of hydrated ${Sc}^{3+}$ is determined to be ${Sc(H_{2}O)}_{8}^{3+}$. On the kaolinite (001) Al-OH surface, it exhibits a preference for adsorption at the deprotonated O_u_ site, achieving stable adsorption through covalent bonding and hydrogen bonding. Among the doped systems, Mg doping exhibits the most pronounced effect, offering the optimal adsorption environment for hydrated ${Sc}^{3+}$, characterized by the most negative adsorption energy and the strongest surface interaction, as evidenced by the highest bond population. Comparatively, the influence of Ca doping on the adsorption behavior is secondary, while Fe doping is detrimental to the adsorption of hydrated ${Sc}^{3+}$, with its adsorption energy even surpassing that of the undoped system. Furthermore, both Mg and Ca doping significantly enhance the covalency of the Al-O bond, leading to a slight shift of the overall density of states towards higher energies, amplifying the contribution of the O 2p orbitals at the Fermi level, thereby enhancing the reactivity of the O atom. Fe doping barely alters the covalency of the Al-O bond, causing a pronounced shift of the overall density of states towards lower energies, with the primary contribution at the Fermi level transitioning from O 2p orbitals to Fe 3d orbitals. These findings provide crucial theoretical guidance for the design of kaolinite and its doped systems in adsorption and catalytic applications.

## Figures and Tables

**Table 1:** Mulliken atomic charge population of ${Sc(H_{2}O)}_{8}^{3+}$, bond lengths of Sc-O, and bond population of the Sc-O bond.

Atom	s	p	d	Total	Charge/e	Sc-O Bond	Bond Length(Å)
O_1_	1.84	5.10	0	6.94	−0.94	0.13	2.191
O_2_	1.84	5.10	0	6.94	−0.94	0.13	2.211
O_3_	1.86	5.10	0	6.96	−0.96	0.13	2.282
O_4_	1.86	5.12	0	6.98	−0.98	0.14	2.322
O_5_	1.82	5.12	0	6.94	−0.94	0.14	2.357
O_6_	1.84	5.12	0	6.96	−0.96	0.13	2.357
O_7_	1.84	5.10	0	6.94	−0.94	0.13	2.181
O_8_	1.86	5.11	0	6.97	−0.97	0.14	2.344
Sc	2.20	5.88	0.88	8.96	2.04	\	\

**Table 2:** Binding energies of doped kaolinite (001) Al-OH surfaces.

Surface	$E_{b}$(eV/Atom)	Atom Energy (eV)		
Mg-doped	−4.311	H	O	Al
Ca-doped	−4.286			
Fe-doped	−4.179	−12.5	−434.36	−52.79

**Table 3:** Adsorption energies and structural parameters of hydrated ${Sc}^{3+}$ on the kaolinite (001) Al-OH surface.

Site	State	N ^a^	R(Sc-O_w_)_avg_^b^/Å	R(Sc-O_s_) ^c^/Å	H_s_-O_w_^d^/Å	H_w_-O_s_^e^/ Å	E_ads/_kJ·mol^−1^
O_u_H	before	7	2.266	1.965	1.383	1.554, 1.606, 1.624, 1.803	−986.56
	After	5	2.175				
O_t_H	before	7	2.270	1.966	1.483, 2.240, 1.992	1.399, 1.564, 1.710	−922.24
	After	5	2.187				
O_l_H	before	7	2.270	1.968	\	1.486, 1.520, 1.554, 1.617, 1.939	−915.04
	After	5	2.182				

^a^ Coordination number between Sc and water molecules. ^b^ Average bond length between Sc and oxygen in water molecules. ^c^ Bond length between Sc and surface oxygen. ^d^ Hydrogen bond length between oxygen in hydrated ${Sc}^{3+}$ and surface H. ^e^ Hydrogen bond length between H in hydrated ${Sc}^{3+}$ and surface oxygen.

**Table 4:** Mulliken atomic and bond populations of hydrated ${Sc}^{3+}$ on the kaolinite (001) surface at the O_u_H site before and after adsorption.

Atomic	s	p	d	Total	Charge (e)	Sc-O_s_ Bond
Sc before	2.18	5.88	0.90	8.96	2.04	0.45
Sc after	2.18	5.90	1.02	9.10	1.90	
O_s_ before	1.87	5.34	0	7.21	−1.21	
O_s_ after	1.86	5.14	0	7.00	−1.00	

**Table 5:** Adsorption energies and structural parameters of hydrated ${Sc}^{3+}$ on the doped kaolinite (001) Al-OH surface.

Site	N	R(Sc-O_w_)_avg_ /Å	R(Sc-O_s_) /Å	H_s_-O_w_ /Å	H_w_-O_s_ /Å	*E_ads_* /kJ·mol^−1^
Pure surface	5	2.175	1.965	1.383	1.554, 1.606, 1.624, 1.803	−986.56
Mg surface	6	2.319	1.873	2.030, 2.230, 2.375, 2.429	1.442, 1.503, 1.704	−1104.16
Ca surface	6	2.310	1.869	\	1.485, 1.523, 1.560, 1.689, 1.759, 2.298	−1088.16
Fe surface	5	2.262	1.971	\	1.470, 1.565, 1.576, 1.653, 1.682	−853.92

**Table 6:** Mulliken atomic and bond populations of Sc and O_s_ after adsorption of hydrated ${Sc}^{3+}$ on the ideal and doped kaolinite surfaces.

State	Sc					O_s_				Sc-O_s_
	s	p	d	Total	Charge	s	p	Total	Charge	
Pure surface	2.18	5.90	1.02	9.10	1.90	1.86	5.14	7.00	−1.00	0.45
Mg surface	2.20	5.88	1.22	9.30	1.70	1.87	5.09	6.96	−0.96	0.60
Ca surface	2.18	5.90	1.21	9.29	1.71	1.85	5.06	6.91	−0.91	0.58
Fe surface	2.20	5.94	1.02	9.16	1.84	1.85	5.05	6.91	−0.91	0.48

## Data Availability

Data are contained within the article.
